# Braden score at ICU admission predicts 30-day mortality in acute pancreatitis

**DOI:** 10.17305/bb.2025.13115

**Published:** 2025-10-15

**Authors:** Lihong Dong, Hong Wang, Xixiang Yang, Xiaolin Zhu, Chen He

**Affiliations:** 1 Department of Critical Care Medicine, The First Affiliated Hospital of Kunming Medical University, Kunming, China; 2 Department of Critical Care Medicine, The Sixth Affiliated Hospital of Kunming Medical University, Yuxi, China; 3 Second Clinical College, Ningxia Medical University, Yinchuan, China

**Keywords:** Braden score, acute pancreatitis, risk of death, MIMIC-IV database

## Abstract

The Braden score, a bedside assessment tool for evaluating the risk of pressure ulcers and frailty, may identify vulnerabilities pertinent to outcomes in acute pancreatitis (AP). However, its prognostic significance in this context remains uncertain. This study aimed to determine whether the Braden score at admission predicts all-cause mortality in intensive care unit (ICU) patients with AP and whether it provides additional value to existing clinical models. In a retrospective single-center cohort study utilizing data from MIMIC-IV v3.1 (2008–2022), we included 1985 adults diagnosed with AP. We analyzed the Braden score as both a continuous variable and a dichotomous variable (high-risk: ≤15 vs low-risk: >15), with 30-day mortality as the primary endpoint (with secondary endpoints at 90, 180, and 360 days). Our methodology encompassed Kaplan–Meier analysis, multivariable Cox regression, restricted cubic splines, receiver operating characteristic curves, and calibration assessments. By the 30-day mark, a total of 230 deaths were recorded (11.6%). Each 1-point increase in the Braden score correlated with a 7.7% reduction in mortality risk (HR 0.923, 95% CI 0.873–0.976; *P* ═ 0.005). Furthermore, patients categorized as low-risk experienced lower mortality rates compared to high-risk patients (HR 0.688, 95% CI 0.501–0.945; *P* ═ 0.021). The discrimination capability at 30 days was moderate (AUC 0.67, 95% CI 0.63–0.71), with an optimal cutoff score of 15 (sensitivity 61%, specificity 65%) and good calibration; however, performance diminished over longer durations. Incorporating the Braden score into a baseline clinical model enhanced predictive accuracy (AUC 0.712 vs 0.647; NRI 0.235; IDI 0.040; all *P* < 0.001). The Braden score at ICU admission is independently associated with 30-day mortality in patients with AP, providing moderate, well-calibrated predictions and significant incremental value. This supports its application as an early and straightforward tool for risk stratification, pending prospective validation.

## Introduction

Acute pancreatitis (AP) is an inflammatory condition of the pancreas characterized by the premature activation of digestive enzymes, leading to self-digestion of pancreatic tissue. As the disease progresses, it can trigger systemic inflammatory responses and potentially result in organ failure [1]. AP is a common gastrointestinal disorder, with an annual incidence ranging from 13 to 45 cases per 100,000 individuals [2]. Over the past two decades, the incidence and hospitalization rates for AP have increased, imposing a significant burden on patients, families, and healthcare systems [3]. The prognosis for AP varies with its severity. Approximately 75%–80% of patients experience a mild course that can be resolved with intravenous fluids and supportive care [4, 5]. In contrast, nearly 20% of patients develop moderate to severe AP, often accompanied by pancreatic or peripancreatic tissue necrosis and organ failure, contributing to an overall mortality rate between 20% and 40% [6, 7]. Consequently, identifying effective prognostic indices to stratify high-risk patients is of critical clinical importance.

Currently, several scoring systems, including the Ranson criteria [8], Balthazar grading [9], APACHE-II [8], Sequential Organ Failure Assessment (SOFA) [10], and the bedside index for severity in AP [11], are employed to predict the severity and prognosis of AP. While these scores enhance the understanding of AP’s progression, they are often complex and time-consuming to implement, which can increase the mortality risk by delaying optimal treatment. Additionally, recent research has identified various biomarkers associated with AP prognosis, such as procalcitonin (PCT), C-reactive protein, interleukin-6, red blood cell distribution width, albumin, creatinine (Cr), blood urea nitrogen (BUN), and serum calcium (Ca) [12–16]. However, the relationship between these individual biomarkers and AP mortality risk remains inadequate due to the complex pathophysiological states of patients [17]. Therefore, there is an urgent need for more straightforward, rapid, highly reproducible, and sensitive indices to assess the all-cause mortality (ACM) risk in AP.

The Braden scale, widely utilized for evaluating pressure ulcer risk [18] and identifying frailty [19], encompasses six dimensions: sensory perception, moisture, mobility, activity, nutritional status, and friction/shear force. Its ease of use and lack of requirement for laboratory data make the Braden score applicable in various medical, surgical, and intensive care contexts [20, 21]. As research has advanced, the Braden score’s applicability has expanded to predict adverse clinical outcomes in critically ill patients, including myocardial infarction, ischemic stroke, delirium, COVID-19, traumatic brain injury, sepsis, and cardiac patients in the intensive care unit (ICU) [22–29]. Although originally designed to assess pressure ulcers, the Braden score’s capacity to evaluate overall frailty has garnered interest for broader clinical applications, particularly given its multidimensional assessments (e.g., mobility and nutritional status), which may be critical in the initiation and progression of AP [30–32]. Nonetheless, no studies have yet established a direct link between Braden scores and ACM risk in AP. Therefore, this study aims to investigate the association between Braden scores and ACM risk, providing a simple, early risk assessment tool for patients with AP, while further elucidating the relationship between mobility/nutritional status in the Braden score and AP prognosis.

## Materials and methods

### Study population

Data for this study were obtained from the MIMIC-IV database (version 3.1), a comprehensive public database developed by the Computational Physiology Laboratory at the Massachusetts Institute of Technology, which contains detailed records of all patients admitted to Beth Israel Deaconess Medical Center from 2008 to 2022 [33]. To protect patient privacy, all personal data were de-identified, with patient identifiers replaced by random codes, thus exempting the study from ethical approval and informed consent requirements. The first author, LhD, completed the Collaborative Institutional Training Initiative course and successfully passed the Conflict of Interest and Data or Specimen Research Only exams (ID: 14326940), obtaining authorization to access the database and extract the necessary variables. This study adhered to the Strengthening the Reporting of Observational Studies in Epidemiology (STROBE) guidelines [34].

**Figure 1.**
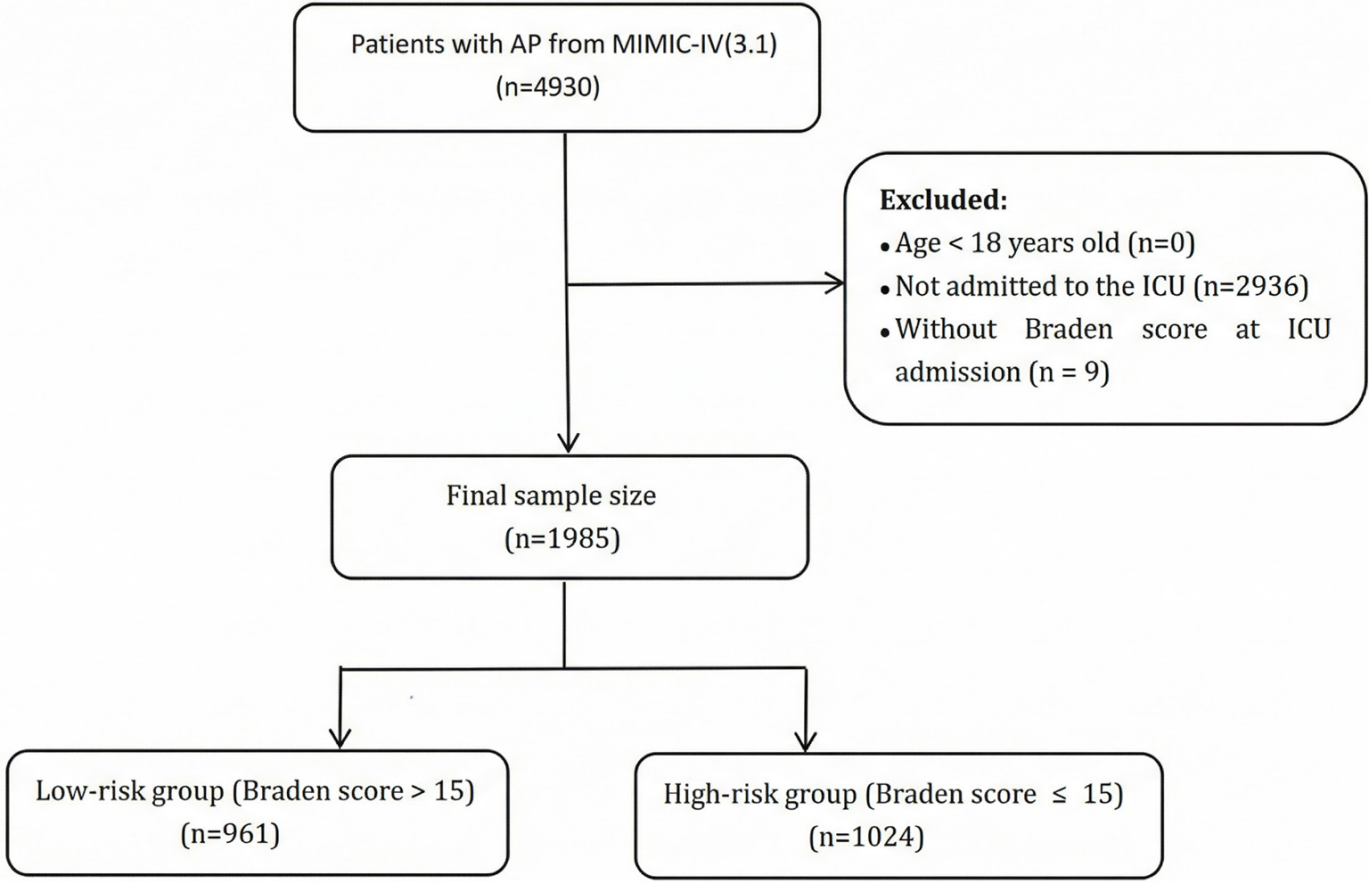
**Flowchart for participants selection**.

ICU admission data for AP patients were collected using the ICD-9 code 577.0 and the ICD-10 codes K85-K85.92. Patients were excluded based on the following criteria: (1) individuals younger than 18 years at the time of initial admission; (2) those not admitted to the ICU; (3) patients with multiple admissions for AP, where only data from the first admission were retained; (4) patients with missing Braden assessment records (Figure 1).

### Braden score assessment

The Braden score, developed in 1987 by American nurses Barbara Braden and Nancy Bergstrom, serves as a widely recognized clinical tool for assessing patients’ risk of pressure ulcers [35]. ICU nurses evaluated the Braden score using the ward’s standardized Pressure Ulcer Risk Screening Form upon patient admission. Prior to assessment, nurses were required to complete online training and pass an accompanying evaluation. In this study, the Braden score at ICU admission served as the exposure factor, encompassing six key components: sensory perception, moisture, mobility, activity, nutritional status, and friction/shear force [36]. Scores for each component ranged from 1 to 4, except for friction/shear, which ranged from 1 to 3. The total score varied from 6 to 23 points, with lower scores indicating a higher risk of pressure ulcers [37]. A cutoff value of 15 was established to categorize participants into low-risk (Braden score > 15) and high-risk groups (≤15) based on clinical expertise and previous literature [25, 29].

### Outcome variables

The primary outcome was the risk of ACM at 30 days, with secondary outcomes including ACM risk at 90 days, 180 days, and 360 days. The time origin for all survival analyses was defined as the date of ICU admission. Patients were monitored from ICU admission until the earliest occurrence of any of the following events: (1) death from any cause; (2) the specified follow-up period (30 days, 90 days, 180 days, or 360 days); or (3) the last recorded entry in the MIMIC-IV database. Death events were identified through the “dod” (date of death) variable within the MIMIC-IV database, which integrated hospital records and state-level death registry data. Patients who survived without recorded deaths during the follow-up period were censored at the earlier conclusion of the follow-up period or their last database entry. This approach ensured consistent identification of both in-hospital and out-of-hospital deaths while minimizing informative censoring due to follow-up loss.

### Data extraction

Data were extracted using PostgreSQL software (version 17) and Navicat Premium software (version 17.2.3) through structured query language. The following variables were collected: 1) demographic data: age, sex, marital status, and ethnicity; 2) vital signs: heart rate and respiratory rate (RR); 3) comorbidities identified based on ICD-9 or ICD-10 codes: mild or severe liver disease, kidney disease, malignant tumors, chronic obstructive pulmonary disease (COPD), congestive heart failure (CHF), peripheral vascular disease (PVD), myocardial infarction (MI), and hypertension (HP); 4) laboratory indicators: WBC, PLT, HGB, HCT, PT, PTT, INR, lactate, albumin, ALT, AST, Cr, BUN, total bilirubin (TB), calcium, and blood glucose (BG); 5) clinical treatment: medications (norepinephrine, statins) and mechanical ventilation (MV); 6) disease scores: Braden score, SOFA score, and Glasgow Coma Scale (GCS). For data with multiple measurements, values recorded on the first day of ICU admission were extracted.

### Methods for outliers and missing value

To mitigate potential bias from sample exclusion, variables with missing values exceeding 20% were eliminated, while those with missing data below 20% were imputed using the random forest imputation method (MissForest) [38]. Outliers were managed using the winsorization method, applying cutoff points at the 1st and 99th percentiles [39, 40].

### Statistical analysis

The normality of continuous variables was assessed using the Shapiro–Wilk test. Continuous variables with a normal distribution were reported as mean ± standard deviation, while skewed distributions were reported as median (interquartile range [IQR]). Normally distributed continuous variables were analyzed using *t*-tests, whereas skewed variables were analyzed with Mann–Whitney *U* tests. Categorical data were presented as percentages (%) and analyzed using the chi-square test or Fisher’s exact test. Patients were classified into high-risk and low-risk groups based on their Braden scores. Survival curves were generated using the Kaplan–Meier (KM) method, with intergroup comparisons conducted using the log-rank test. Cox regression models were employed to examine the association between Braden scores and outcomes, providing hazard ratios (HR) and 95% confidence intervals (CIs). Three models were constructed: Model 1 (unadjusted), Model 2 (adjusted for age, sex, marital status, and race), and Model 3 (considering demographic information, vital signs, laboratory indicators [albumin, AST, BUN, lactate, Ca, Cr, BG, HCT, PLT, PT, PTT, TB, WBC], comorbidities, clinical treatment, and GCS score). To address multicollinearity, the variance inflation factor (VIF) was calculated for each variable, excluding those with a VIF > 5. Variables including ALT (VIF = 6.02), HGB (VIF = 24.15), and INR (VIF = 25.04) were excluded. Although the initial VIFs for AST, HCT, and PT exceeded 5, they subsequently decreased to 1.63, 1.34, and 1.83, respectively, indicating that their high correlations were primarily influenced by the excluded variables (Figures S1 and S2). Restricted cubic spline (RCS) curves were utilized to investigate the potential linear relationship between Braden scores and ACM risk, with three nodes corresponding to the 10th, 50th, and 75th percentiles of the Braden score, using ICU admission as the time origin. Receiver operating characteristic (ROC) analysis was conducted to evaluate the predictive power of Braden scores for ACM risk at 30, 90, 180, and 360 days post-ICU admission, determining sensitivity and specificity, and calculating the area under the curve (AUC). The net reclassification improvement (NRI) and integrated discrimination improvement (IDI) were computed to assess the additional predictive value of the Braden scale for ACM risk in AP patients. A calibration curve was generated to evaluate the consistency between model predictions and actual observations. Subgroup analyses were performed to explore relationships within different demographic groups: age, sex, marital status, ethnicity, mild or severe liver disease, kidney disease, malignant tumors, COPD, CHF, PVD, MI, HP, norepinephrine, statins, and MV. The log-likelihood ratio test was utilized to assess interactions between the Braden score and other variables. All data processing, analyses, and graphical representations were conducted using R software version 4.4.3. A *P* value < 0.05 was deemed statistically significant.

**Table 1:** Baseline characteristics of the study population

**Variable**	**Overall (*n* ═ 1985)**	**High-risk group^a^** **(*n* ═ 1024)**	**Low-risk group (*n* ═ 961)**	***P***
*Personal characteristics*				
Age_group (%)				<0.001*
<60	1004 (50.6)	477 (46.6)	527 (54.8)	
>=60	981 (49.4)	547 (53.4)	434 (45.2)	
Gender (%)				0.76
Male	1126 (56.7)	577 (56.3)	549 (57.1)	
Female	859 (43.3)	447 (43.7)	412 (42.9)	
Marital (%)				0.003*
Single	632 (31.8)	326 (31.8)	306 (31.8)	
Divorced/Widowed	333 (16.8)	162 (15.8)	171 (17.8)	
Married	851 (42.9)	426 (41.6)	425 (44.2)	
Unknown	169 (8.51)	110 (10.7)	59 (6.14)	
Race (%)				<0.001*
White	1258 (63.4)	641 (62.6)	617 (64.2)	
No White	509 (25.6)	237 (23.1)	272 (28.3)	
Unknown	218 (11.0)	146 (14.3)	72 (7.49)	
*Vital signs and laboratory tests*				
Heart rate (beats/min)	94.0 [80.0;110]	94.0 [80.0;111]	93.0 [81.0;109]	0.236
Respiration rate (beats/min)	19.0 [16.0;24.0]	19.5 [16.0;24.0]	19.0 [16.0;23.0]	0.393
Albumin (g/dL)	3.10 [2.60;3.60]	2.90 [2.50;3.42]	3.30 [2.80;3.80]	<0.001*
ALT (IU/L)	38.0 [19.0;94.0]	38.0 [19.0;102]	38.0 [20.0;88.0]	0.431
AST (IU/L)	52.0 [27.0;132]	58.0 [29.0;150]	48.0 [25.0;115]	<0.001*
BUN (mg/dL)	18.0 [12.0;33.0]	21.0 [13.0;37.0]	17.0 [11.0;29.0]	<0.001*
Calcium (mg/dL)	8.10 [7.60;8.70]	8.00 [7.40;8.60]	8.20 [7.70;8.80]	<0.001*
Creatinine (mg/dL)	1.00 [0.70;1.70]	1.10 [0.70;1.80]	0.90 [0.70;1.50]	<0.001*
Glucose (mg/dL)	126 [102;167]	129 [103;174]	123 [99.0;158]	0.002*
HGB (g/dL)	10.7 [9.10;12.3]	10.5 [9.00;12.1]	11.0 [9.30;12.5]	<0.001*
HCT (%)	32.4 [27.7;37.1]	32.0 [27.4;36.8]	33.0 [28.2;37.7]	0.003*
INR	1.30 [1.10;1.60]	1.30 [1.20;1.60]	1.20 [1.10;1.50]	<0.001
Lac (mmol/L)	1.80 [1.30;2.70]	1.90 [1.30;2.90]	1.70 [1.20;2.50]	<0.001
PLT (K/uL)	185 [126;260]	179 [121;256]	191 [137;262]	0.017
PT (s)	14.3 [12.6;17.1]	14.7 [13.0;17.6]	13.7 [12.3;16.4]	<0.001
PTT (s)	30.6 [27.2;36.8]	31.3 [27.6;38.4]	29.8 [26.9;34.9]	<0.001
Total bili (mg/dL)	0.80 [0.40;2.20]	0.90 [0.50;2.42]	0.80 [0.40;2.00]	0.001
WBC (K/uL)	11.0 [7.40;16.3]	11.9 [8.10;17.4]	10.2 [6.90;15.3]	<0.001
*Comorbidities*				
Mild liver disease (%)				0.374
No	1235 (62.2)	627 (61.2)	608 (63.3)	
Yes	750 (37.8)	397 (38.8)	353 (36.7)	
Renal disease (%)				0.72
No	1367 (68.9)	701 (68.5)	666 (69.3)	
Yes	618 (31.1)	323 (31.5)	295 (30.7)	
Severe liver disease (%)				0.111
No	1653 (83.3)	839 (81.9)	814 (84.7)	
Yes	332 (16.7)	185 (18.1)	147 (15.3)	
Malignant cancer (%)				0.531
No	1609 (81.1)	836 (81.6)	773 (80.4)	
Yes	376 (18.9)	188 (18.4)	188 (19.6)	
Chronic pulmonary disease (%)				0.682
No	1373 (69.2)	713 (69.6)	660 (68.7)	
Yes	612 (30.8)	311 (30.4)	301 (31.3)	
Congestive heart failure (%)				0.797
No	1378 (69.4)	714 (69.7)	664 (69.1)	
Yes	607 (30.6)	310 (30.3)	297 (30.9)	
Peripheral vascular disease (%)				0.97
No	1650 (83.1)	852 (83.2)	798 (83.0)	
Yes	335 (16.9)	172 (16.8)	163 (17.0)	
Myocardial infarct (%)				0.791
No	1624 (81.8)	835 (81.5)	789 (82.1)	
Yes	361 (18.2)	189 (18.5)	172 (17.9)	
Hypertension (%)				0.469
No	794 (40.0)	418 (40.8)	376 (39.1)	
Yes	1191 (60.0)	606 (59.2)	585 (60.9)	
Norepinephrine (%)				<0.001*
No	1323 (66.6)	608 (59.4)	715 (74.4)	
Yes	662 (33.4)	416 (40.6)	246 (25.6)	
Statins (%)				0.719
No	945 (47.6)	492 (48.0)	453 (47.1)	
Yes	1040 (52.4)	532 (52.0)	508 (52.9)	
Mechanical ventilation (%)				<0.001*
No	1054 (53.1)	397 (38.8)	657 (68.4)	
Yes	931 (46.9)	627 (61.2)	304 (31.6)	
*Scores*				
GCS	15.0 [15.0;15.0]	15.0 [14.0;15.0]	15.0 [15.0;15.0]	<0.001*
SOFA	5.00 [2.00;8.00]	6.00 [3.00;9.00]	4.00 [2.00;6.00]	<0.001*

^a^In our study, the low-risk group was defined as a Braden score > 15 and the high-risk group was defined as a Braden score ≤ 15. ^*^Significant difference between two groups (*P* < 0.05). Abbreviations: GCS: Glasgow Coma Scale; SOFA: Sequential organ failure assessment; ALT: Alanine aminotransferase; AST: Aspartate aminotransferase; HGB: Hemoglobin; HCT: Hematocrit; PLT: Platelet count; PT: Prothrombin time; PTT: Partial thromboplastin time; WBC: White blood cell count.

### Declaration on exploratory analysis

All subgroup analyses and comparisons of long-term endpoints at 90, 180, and 360 days were exploratory in nature. No adjustments were made for multiple testing. These results were intended solely to generate hypotheses and identify potential signals and should not be regarded as definitive conclusions. Further validation in independent prospective cohorts is required.

## Results

### Baseline traits

According to the established criteria, 1985 AP patients were included. The basic clinical traits are outlined in Table 1. The high-risk group mainly consisted of older people and Caucasians (*P* < 0.001). Additionally, high-risk populations had lower initial laboratory values for ALB, HGB, HCT, PLT, and Ca levels at admission, while AST, BUN, lactate, Cr, BG, INR, PT, PTT, TB, and WBC levels were higher. Furthermore, high-risk patients exhibited higher SOFA scores (*P* < 0.001), indicating more severe illness, and were more likely to require norepinephrine support and MV (*P* < 0.001). No prominent differences were discerned in sex, heart rate, RR, ALT, mild or severe liver disease, kidney disease, malignant tumors, COPD, CHF, PVD, MI, HP, and statins (*P* > 0.05).

### KM survival curve

**Figure 2.**
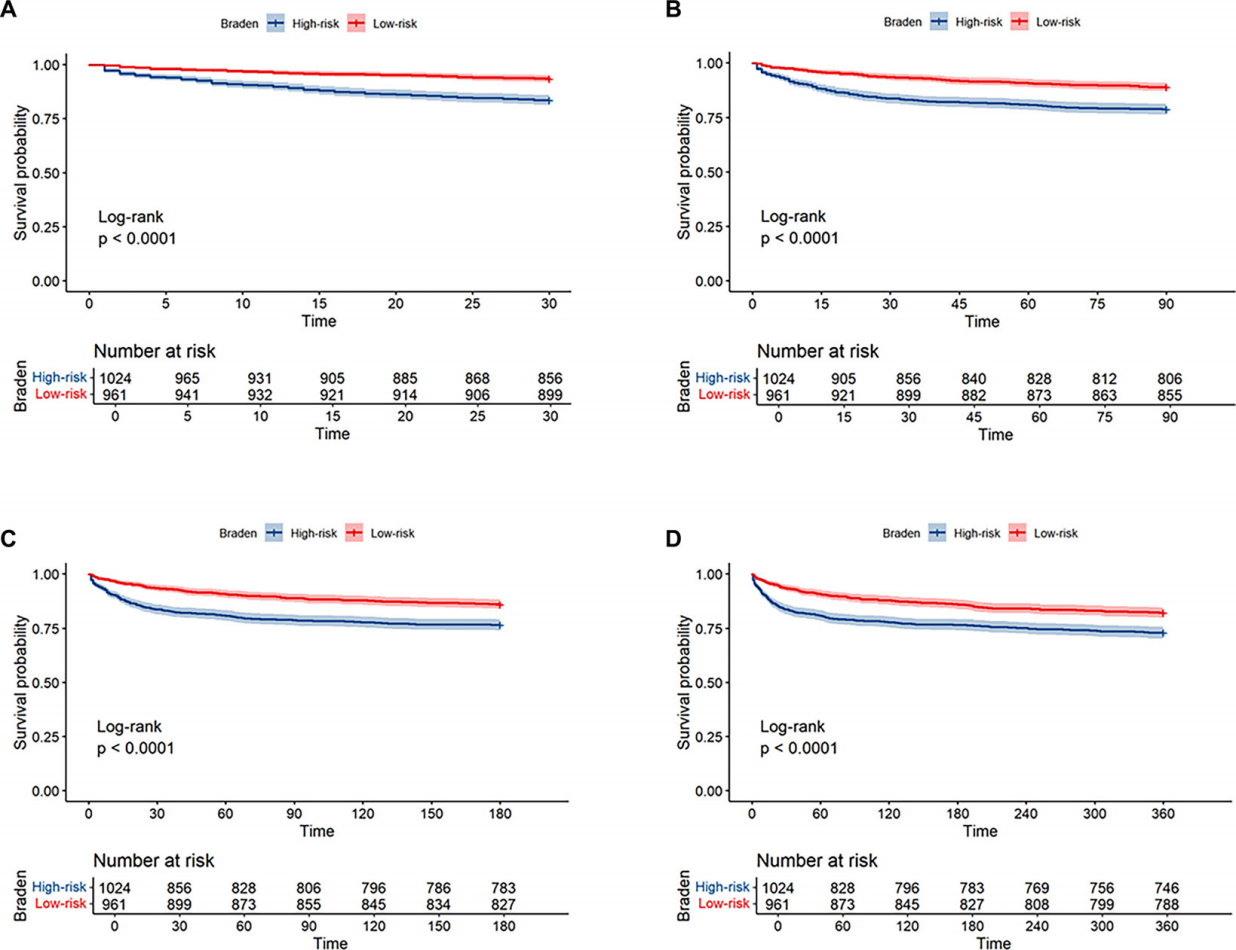
**Kaplan–Meier survival curves for ACM by Braden risk category in AP.** In the overall cohort (*n* ═ 1985), 230, 324, 375, and 451 deaths occurred within 30, 90, 180, and 360 days, respectively. At each time point, high-risk patients (Braden score ≤ 15) had higher ACM than low-risk patients (Braden score > 15); all log-rank *P* < 0.001. (A) 30-day; (B) 90-day; (C) 180-day; and (D) 360-day mortality. Numbers at risk are shown beneath each plot. Abbreviations: AP: Acute pancreatitis; ACM: All-cause mortality.

Among the 1985 AP patients, 230 died within 30 days, 324 within 90 days, 375 within 180 days, and 451 within 360 days. The KM curve demonstrated significant differences in ACM risk between the high-risk and low-risk groups at 30 days, 90 days, 180 days, and 360 days (Figure 2). High-risk patients exhibited a greater ACM risk than low-risk patients at these time points (all log-rank *P* < 0.001).

**Figure 3.**
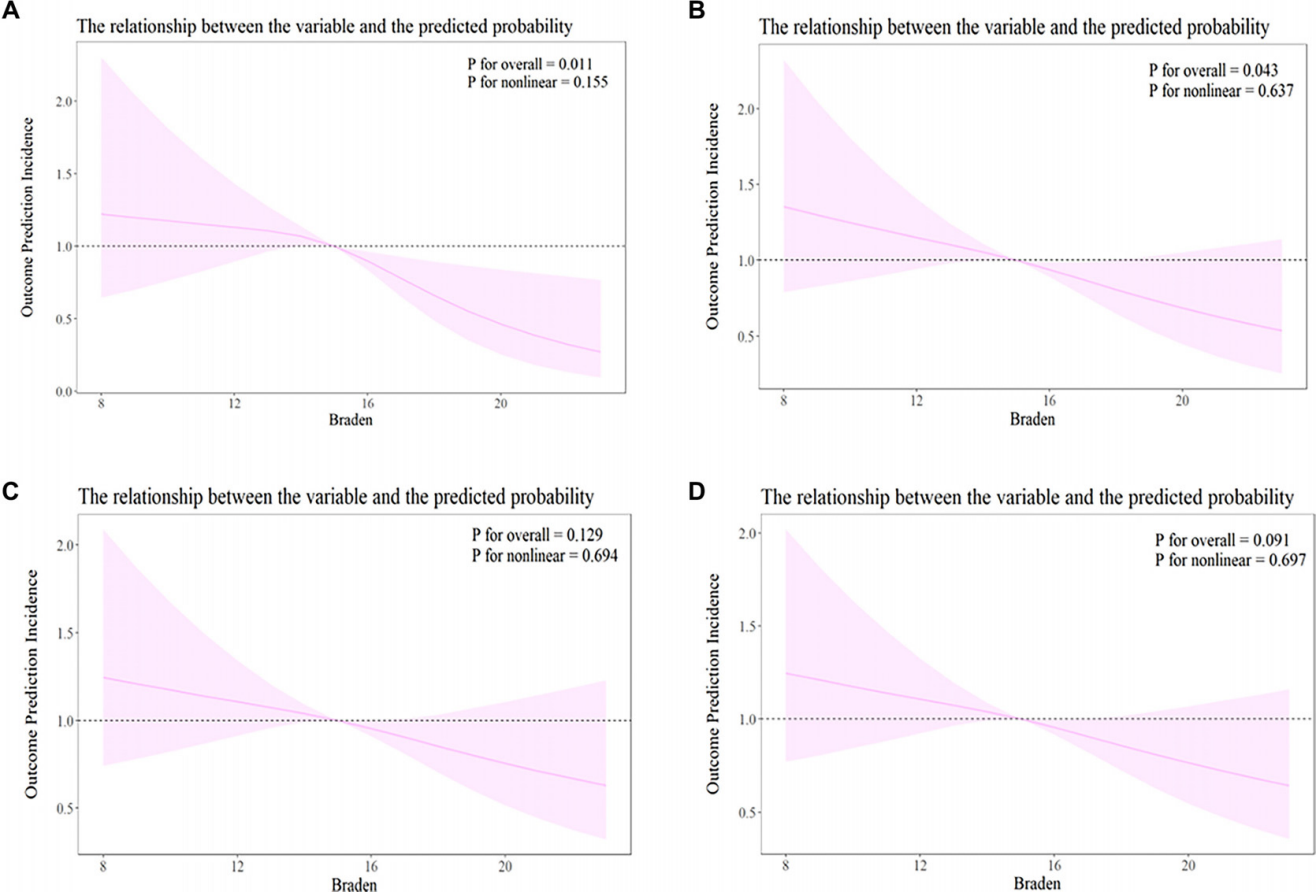
**RCS models showing the dose–response relationship between admission Braden score and all-cause mortality risk in acute pancreatitis patients at (A) 30-day, (B) 90-day, (C) 180-day, and (D) 360-day follow-up.** The black dashed line represents the HR, with the shaded area indicating the 95% confidence interval. Abbreviations: RCS: Restricted cubic spline; HR: Hazard ratio.

### Relationship between the braden scale and prognosis in AP patients

Cox regression models indicated that each 1-unit increase in the Braden score, when treated as a continuous variable, was associated with a significantly reduced ACM risk. Specifically, for the 30-day ACM risk, the HR and 95% CIs across three models were as follows: 0.81 (0.772–0.851), 0.822 (0.781–0.866), and 0.923 (0.873–0.976) (all *P* < 0.05). Similar results were observed for 90-day, 180-day, and 360-day ACM risk. When the Braden score was evaluated as a dichotomous variable, the low-risk group was significantly associated with reduced 30-day ACM risk compared to the high-risk group (Model 1: HR, 0.376 [95% CI 0.282–0.502] *P* < 0.001; Model 2: HR, 0.422 [95% CI 0.315–0.565] *P* < 0.001; Model 3: HR, 0.688 [95% CI 0.501–0.945] *P* ═ 0.021). However, after adjusting for all confounders, no significant association was identified between Braden scores and ACM risk at 90 days, 180 days, and 360 days (Table 2). The HRs and CIs for each confounding factor are presented in Figure S3.

**Table 2:** Univariate and multivariate Cox regression models of Braden score with mortality in patients with acute pancreatitis

**Outcome**	**Model 1**			**Model 2**			**Model 3**		
	**HR**	**95% CI**	***P***	**HR**	**95% CI**	***P***	**HR**	**95% CI**	***P***
*30-day mortality*									
Continuous	0.810	0.772–0.851	<0.001	0.822	0.781–0.866	<0.001	0.923	0.873–0.976	0.005
*Category*									
High-risk (Braden score ≤ 15)	Ref.	Ref.		Ref.	Ref.		Ref.	Ref.	
Low-risk (Braden score > 15)	0.376	0.282–0.502	<0.001	0.422	0.315–0.565	<0.001	0.688	0.501–0.945	0.021
*90-day mortality*									
Continuous	0.848	0.814–0.883	<0.001	0.858	0.823–0.895	<0.001	0.943	0.901–0.988	0.013
*Category*									
High-risk (Braden score ≤ 15)	Ref.	Ref.		Ref.	Ref.		Ref.	Ref.	
Low-risk (Braden score > 15)	0.482	0.382–0.607	<0.001	0.530	0.419–0.670	<0.001	0.791	0.614–1.019	0.07
*180-day mortality*									
Continuous	0.869	0.837–0.902	<0.001	0.881	0.847–0.916	<0.001	0.957	0.918–0.999	0.045
*Category*									
High-risk (Braden score ≤ 15)	Ref.	Ref.		Ref.	Ref.		Ref.	Ref.	
Low-risk (Braden score > 15)	0.549	0.445–0.678	<0.001	0.607	0.491–0.751	<0.001	0.875	0.694–1.103	0.3
*360-day mortality*									
Continuous	0.884	0.855–0.915	<0.001	0.893	0.862–0.925	<0.001	0.958	0.922–0.996	0.03
*Category*									
High-risk (Braden score ≤ 15)	Ref.	Ref.		Ref.	Ref.		Ref.	Ref.	
Low-risk (Braden score > 15)	0.607	0.502–0.734	<0.001	0.658	0.543–0.797	<0.001	0.905	0.734–1.114	0.3

Model 1: Unadjusted; Model 2: Adjusted for admission age group, gender, marital, race; Model 3: Adjusted for admission age group, gender, marital, race, heart rate, RR, albumin, aspartate transaminase, urea nitrogen, calcium, creatinine, glucose, hematocrit, lactate, platelet, PT, PTT, total bilirubin, WBC, MLD, renal disease, severe liver disease, malignant cancer, COPD, congestive heart failure, peripheral vascular disease, myocardial infarct, HP, norepinephrine, statins, MV, GCS. Abbreviations: HR: Hazard ratio; CI: Confidence interval; RR: Respiratory rate; PT: Prothrombin time; PTT: Partial thromboplastin time; WBC: White blood cell; MLD: Mean lung density; COPD: Chronic obstructive pulmonary disease; HP: Hypertension; MV: Mechanical ventilation; GCS: Glasgow Coma Scale.

RCS analysis (Figure 3) revealed that Braden scores exhibited a significant linear relationship with ACM risk at 30 days (*P* for nonlinear = 0.155) and 90 days (*P* for nonlinear = 0.637). However, no nonlinear associations were found at 180 and 360 days, although the Braden score demonstrated a significant linear protective trend at both 180 and 360 days.

### Prognostic value of braden scores for AP patients

**Figure 4.**
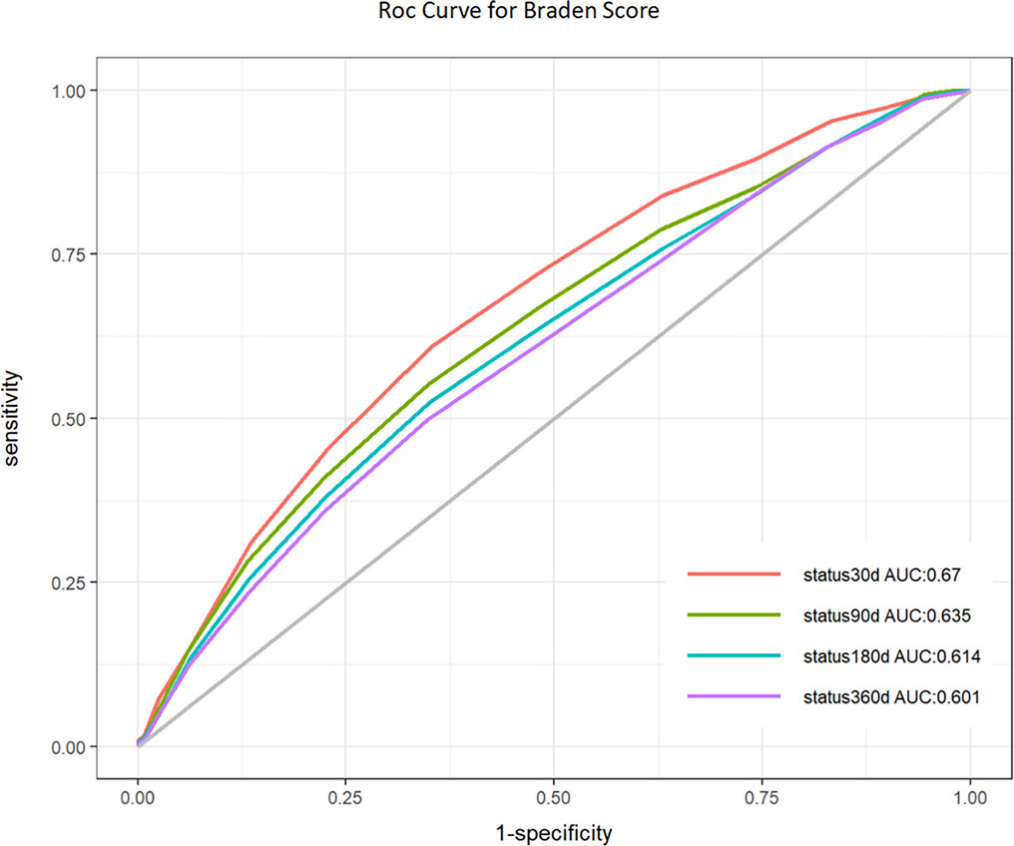
**ROC curve for Braden Scale’s predictive accuracy.** ROC curve demonstrating the Braden Scale’s efficacy in predicting 30-day mortality, 90-day mortality, 180-day mortality, 1-year mortality, with the calculated AUC. Abbreviations: ROC: Receiver operating characteristic; AUC: Area under the curve.

**Figure 5.**
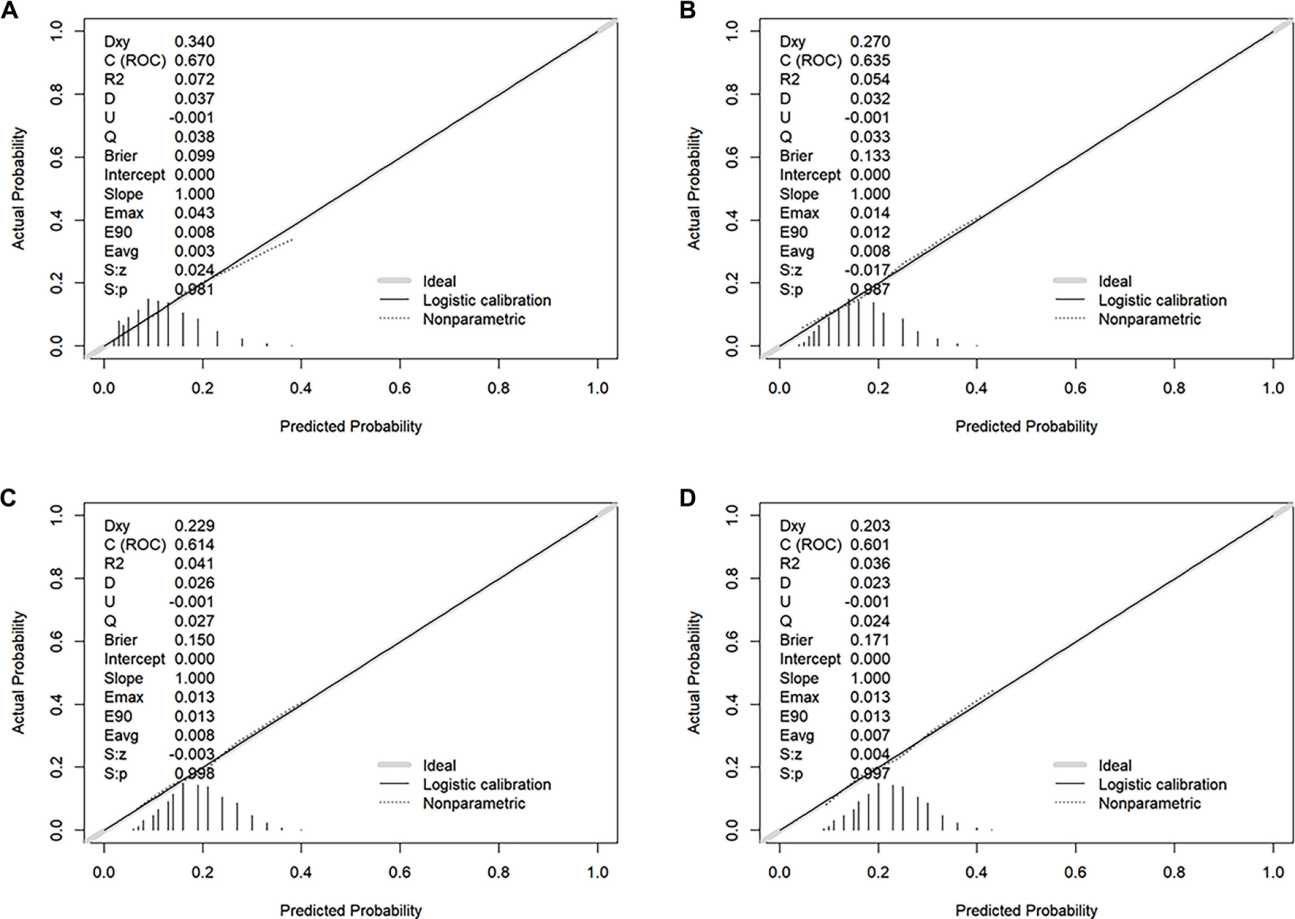
**Calibration of the Braden score for all-cause mortality in AP at (A) 30, (B) 90, (C) 180, and (D) 360 days.** Predicted probabilities closely matched observed probabilities, exhibiting no significant systematic deviation. Abbreviations: ROC: Receiver operating characteristic; AP: Acute pancreatitis.

ROC curves (Figure 4, Table 3) indicated that the Braden score provided significant predictive value, with a 30-day AUC of 67.02% (95% CI: 63.44–70.61), significantly superior to the AUCs of 63.51% (95% CI: 60.22–66.81) at 90 days, 61.43% (95% CI: 58.29–64.57) at 180 days, and 60.13% (95% CI: 57.18–63.07) at 360 days. Additionally, the optimal cutoff value for the Braden score was determined to be 15, achieving the highest sensitivity (61.04%) and specificity (64.71%) at 30 days. The Braden score also demonstrated good calibration in predicting 30-day mortality risk, aligning predicted probabilities closely with observed probabilities without significant systematic deviation (Figure 5). This supports the favorable predictive capability of the Braden score for ACM risk in AP patients, underscoring its clinical utility. Incorporating the Braden score into Model 2 (which included conventional variables such as age, sex, marital status, and ethnicity) resulted in an increased AUC, with this increase being statistically significant (Table 4). To evaluate the model’s ability to reclassify risk, the NRI and IDI were calculated. The inclusion of the Braden score enhanced the NRI for Model 2 and improved the IDI (Table 4), suggesting that incorporating the Braden score may enhance the predictive model’s accuracy and risk reclassification capabilities.

**Table 3:** Information of ROC curves

**Variables**	**AUC (%)**	**95% CI**	**Threshold**	**Specificity**	**Sensitivity**
Status 30d	67.02	63.44–70.61	15	0.6471	0.6104
Status 90d	63.51	60.22–66.81	15	0.6504	0.5521
Status 180d	61.43	58.29–64.57	15	0.6501	0.5239
Status 360d	60.13	57.18–63.07	15	0.6512	0.4989

Abbreviations: ROC: Receiver operating characteristic; AUC: Area under the curve; CI: Confidence interval.

**Table 4:** The performance indicators of multivariate models (including the Braden model and the model without the Braden component) in predicting the all-cause mortality risk of AP patients

	**AUC**		**Net reclassification improvement**		**Integrated discrimination improvement**	
	**Index (95% CI)**	***P* value for Δ AUC**	**Index (95% CI)**	***P* value**	**Index (95% CI)**	***P* value**
30d mortality with Braden	0.712 (0.675–0.749)	*P* < 0.001	0.235 (0.161–0.291)	*P* < 0.001	0.040 (0.023–0.064)	*P* < 0.001
30d mortality without Braden	0.647 (0.609–0.685)					
90d mortality with Braden	0.687 (0.654–0.719)	*P* < 0.001	0.193 (0.128–0.248)	*P* < 0.001	0.034 (0.018–0.054)	*P* < 0.001
90d mortality without Braden	0.643 (0.611–0.676)					
180d mortality with Braden	0.683 (0.653–0.714)	*P* < 0.001	0.160 (0.100–0.209)	*P* < 0.001	0.027 (0.013–0.045)	*P* < 0.001
180d mortality without Braden	0.651 (0.620–0.681)					
360d mortality with Braden	0.666 (0.637–0.695)	*P* < 0.001	0.134 (0.081–0.186)	*P* < 0.001	0.025 (0.012–0.041)	*P* < 0.001
360d mortality without Braden	0.636 (0.607–0.665)					

Abbreviations: AUC: Area under the receiver operating characteristic curve; CI: Confidence interval.

### Subgroup analyses

**Figure 6.**
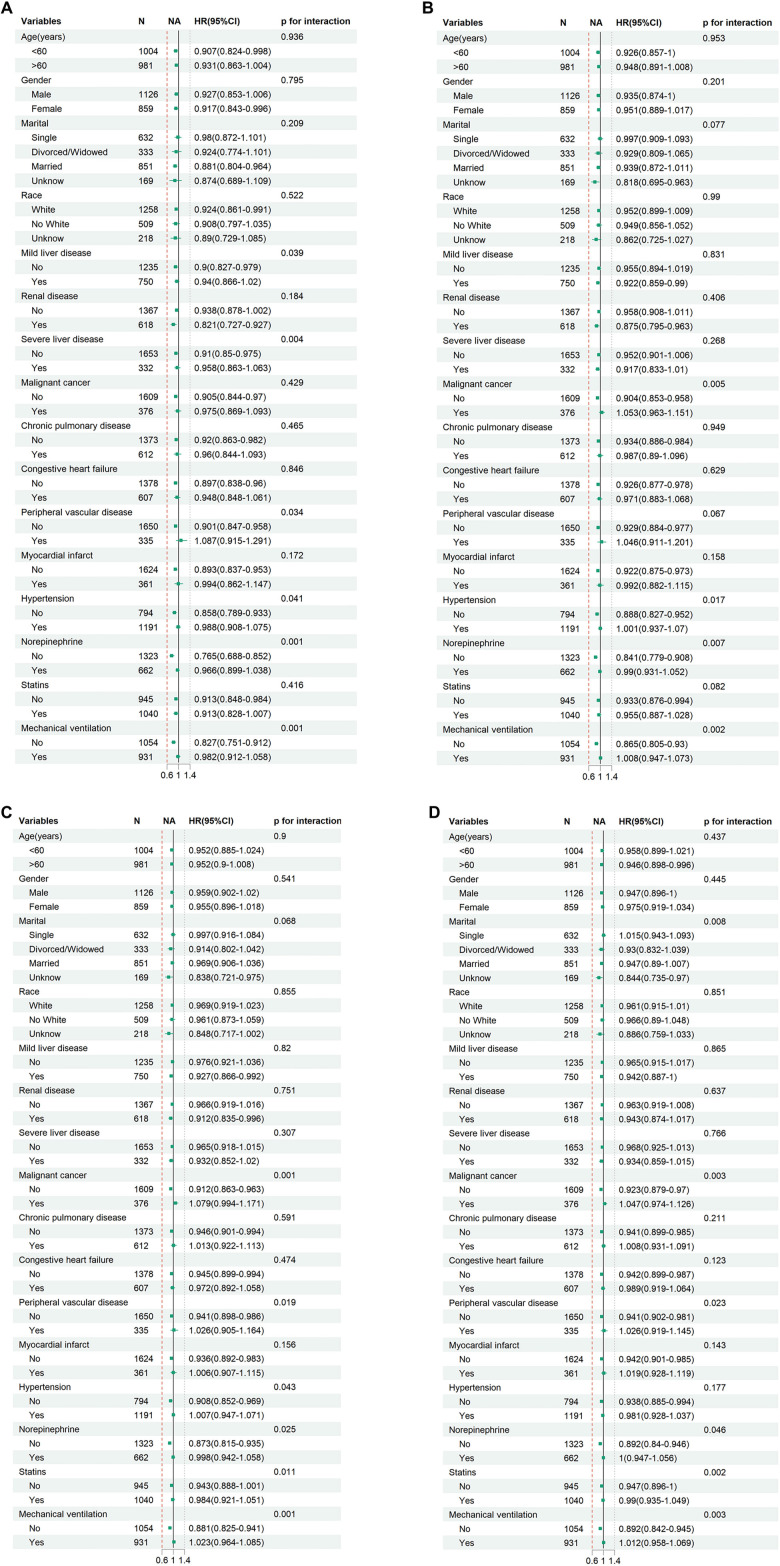
**Forest plots showing subgroup analyses of the association between admission Braden score (low-risk: >15 vs high-risk: ≤15) and all-cause mortality in acute pancreatitis patients at (A) 30-day, (B) 90-day, (C) 180-day, and (D) 360-day follow-up.** Hazard ratios (HRs) and 95% confidence intervals (CIs) were derived from Cox proportional hazards models adjusted for relevant covariates. Subgroups were stratified by demographic and clinical characteristics. Interaction *P* values between Braden score and each variable are shown. All subgroup comparisons are exploratory and were not adjusted for multiple testing.

We further examined the potential association between the Braden score and ACM risk at 30, 90, 180, and 360 days across different cohorts of AP patients. Stratified analyses by age, sex, marital status, race, and comorbidities indicated potential associations between the Braden score and 30-day mortality risk in subgroups of patients aged <60 years, females, Caucasians, married individuals, and those with renal disease (none adjusted for multiple comparisons). Additionally, the Braden score exhibited interaction effects with mild liver disease, severe liver disease, peripheral vascular disease, hypertension, norepinephrine use, and mechanical ventilation (*P* < 0.05), although these findings remain exploratory. For long-term endpoints at 90, 180, and 360 days, only malignant tumors, norepinephrine, and mechanical ventilation showed potential interaction signals (*P* < 0.05). Figure 6 illustrates these hypothesis-generating findings, which require further validation in independent cohorts.

### Sensitivity analysis

To validate the robustness of the strategy for handling missing values, we conducted a sensitivity analysis: 1. After excluding variables with a missing rate >20%, the analysis of complete cases without imputation (*n* ═ 1585, 79.8%) showed that each 1-point increase in the Braden score was associated with a 30-day mortality HR of 0.931 (95% CI 0.870–0.996, *P* ═ 0.037), consistent with the primary estimate (Table S1); 2. After excluding variables with a missing rate >10%, the remaining variables with a missing rate ≤10% (31 variables in total) were imputed using the MissForest algorithm, followed by the primary Cox regression model. Results indicated that the HR for 30-day mortality associated with the Braden score was 0.909 (95% CI 0.861–0.959, *P* < 0.001), which was highly consistent with the results from full text imputation (Table S2). This indicates that the primary conclusions are robust to imputation strategies and missing data proportions.

## Discussion

This study represents the first cohort analysis investigating the relationship between Braden scores at admission and outcomes in AP. Utilizing a large public medical database, we conducted a retrospective analysis. Our findings indicate that the Braden score serves as an independent predictor of 30-day ACM risk in patients with AP, with this significance persisting even after adjusting for potential confounders. We found a linear correlation between the Braden score and ACM risk in AP patients. KM survival analysis confirmed that high-risk patients exhibited increased ACM risk at 30, 90, 180, and 360 days. Additionally, the Braden score demonstrated robust predictive capabilities for ACM risk in AP, with higher AUC values at 30 days compared to 90, 180, and 360 days. Subgroup analyses further validated these results. Thus, this study highlights the Braden score as an early, simple, and efficient tool for assessing ACM risk in AP patients.

Research on the association between the Braden Scale and disease outcomes in ICUs has garnered increasing attention. For instance, Ding et al. reported a strong association between the Braden score and mortality risk in critically ill septic patients [27]. Tang et al. [23] found that the Braden score effectively predicted 30-day mortality risk in critically ill patients with ischemic stroke, achieving an AUC of 0.71. Shang et al. [41] demonstrated that a Braden score below 16 could predict delirium risk in critically ill surgical patients. Yang et al. [42] further emphasized the significant correlation between the Braden score and ACM risk in critically ill individuals with non-traumatic subarachnoid hemorrhage. Consistently, our findings underscore the potential utility of Braden scores in evaluating the prognosis of AP and elucidate the connection between Braden scores and pancreatitis outcomes.

Traditionally, the Braden score is recognized as an effective metric for assessing patients’ risk of pressure ulcers. Our study further extends its applicability to evaluating ACM risk in AP patients. The Braden score’s significance may be attributed to its comprehensive assessment of a patient’s overall health across six dimensions. A lower Braden score typically indicates a higher risk and greater issues in these areas. Several mechanisms may clarify this association. First, patients with moisture exposure, sensory impairment, and reduced mobility are more likely to be bedridden for extended periods, increasing their risk of pressure ulcers and deep vein thrombosis (DVT). An international study involving 1117 ICU wards confirmed a strong correlation between low Braden scores and pressure ulcer incidence, with mortality risk escalating as pressure ulcer severity increased [43]. Additionally, Suryawanshi et al. reported a high incidence of limb DVT in AP patients [44], potentially due to prolonged bed rest and inflammatory cascades [45]. The mechanisms underlying venous thrombosis involve reduced venous return pressure, a hypercoagulable blood state, and systemic inflammatory responses, which can result in vascular endothelial damage [46]. Thrombosis is closely linked to the severity of AP, and the combination of thrombosis and inflammatory biomarkers may aid in predicting short-term outcomes in AP patients [47]. Moreover, nutritional status, a key dimension of the Braden score, is crucial as AP patients experience high catabolism, leading to significant protein and glycogen depletion, malnutrition, and impaired immune function, thereby increasing susceptibility to infections and inflammatory responses, ultimately raising mortality risk [48]. Malnutrition can also alter the intestinal epithelial barrier function and increase mucosal permeability, leading to bacterial translocation, pancreatic tissue necrosis, infections, and multiple organ dysfunction syndrome (MODS) [49]. Furthermore, prolonged bed rest, impaired motor function, malnutrition, and persistent inflammation in AP patients can result in significant muscle wasting. Multiple studies have identified sarcopenia as a poor prognostic factor for AP, increasing mortality risk among ICU-admitted AP patients [50–52]. As previously mentioned, the Braden score offers a comprehensive approach that integrates functional and nutritional aspects to assess patient status from multiple perspectives, making it a valuable bedside tool for identifying mortality risk in AP patients, facilitating early clinical intervention, and improving prognosis.

The pathophysiological mechanisms of AP are complex, involving the autoactivation of pancreatic enzymes, oxidative stress, and immune dysregulation, leading to the release of damage-associated molecular patterns (DAMPs). This initiates an inflammatory cascade that can ultimately culminate in systemic inflammatory response syndrome (SIRS) and MODS [53–55]. Patients with low Braden scores often exhibit reduced mobility and malnutrition, which may exacerbate oxidative stress and immune dysregulation, intensifying pancreatic inflammatory responses and triggering MODS, consequently increasing mortality risk. However, it is important to note that this study demonstrates only statistical associations; causal pathways require validation through prospective cohort or experimental studies.

Our research found that the Braden score is a robust predictor of 30-day mortality, but its predictive capacity declines over longer periods (90, 180, and 360 days). This decline is likely due to the Braden score reflecting the immediate frailty status at admission, which is closely associated with early hospital complications such as pressure ulcers, DVT, and hospital-acquired infections. Therefore, the prediction of 30-day mortality is reliable. In contrast, once patients enter the chronic phase, long-term mortality becomes increasingly dependent on dynamic factors such as pancreatic necrosis infections, recurrent exacerbations, new-onset diabetes or exocrine insufficiency, cardiovascular events, persistent inflammation, and progression of sarcopenia [56]. Consequently, the predictive power of the Braden score diminishes over time. Clinically, combining the Braden score with indicators that can be reassessed 3–6 months post-discharge (e.g., SOFA trend, CRP/albumin ratio, HbA1c, residual necrosis on imaging, gait speed, or handgrip strength) and chronic disease burden (e.g., frailty index and readmission frequency) in a joint model may enhance long-term predictive accuracy.

Exploratory subgroup analyses indicated that the association between Braden scores and 30-day mortality was notably stronger in patients under 60 years of age, females, and individuals with chronic kidney disease (Figure 6). These findings are purely hypothesis-generating and have not undergone multiple corrections, necessitating validation in external cohorts. One possible explanation for these observations is that the baseline organ reserve in these populations has not yet been depleted by advanced age or severe comorbidities, allowing nutritional-functional status to significantly impact short-term outcomes. Younger and female patients may experience a more rapid decline in muscle mass and immune reserve, making the nutritional and activity deficits indicated by low Braden scores more likely to result in early adverse events. Chronic kidney disease often correlates with protein depletion, anemia, and immunosuppression, which overlap significantly with the nutritional and friction-shear dimensions of the Braden scale, potentially enhancing its sensitivity. Conversely, in critically ill patients with decompensated liver disease, we observed a reduced discriminatory effect of the Braden score. We hypothesize that the coexistence of pancreatitis and severe liver disease may lead to pancreatic enzymes entering the liver via the portal vein, exacerbating hepatic injury and triggering systemic inflammatory responses [57, 58]. Additionally, factors such as hypoalbuminemia, ascites, and hepatic encephalopathy may result in consistently low Braden scores within the nutrition/hydration subscale, thereby diminishing its additional discriminatory value. Similarly, in critically ill patients requiring mechanical ventilation or norepinephrine support, this association nearly disappeared, suggesting that once patients enter a state of overt multiple organ failure, baseline frailty indicators may be overshadowed by the extreme severity of their condition. Consequently, organ failure itself, rather than skin-activity risk, predominates short-term prognosis, thus diminishing the discriminatory power of the Braden scale [59, 60]. Therefore, a Braden score of ≤15 has limited value as a standalone alert threshold in populations requiring intensive organ support. In clinical practice, it should be utilized in conjunction with dynamic indicators such as SOFA and lactate for a comprehensive assessment. For patients with mild-to-moderate AP or those in the aforementioned high-risk subgroups, the Braden score may function as a straightforward, early risk stratification tool.

One of the primary strengths of this study is its initial proposition that the Braden score is an independent predictor of ACM risk in AP. The MIMIC-IV database provides extensive and diverse population data, allowing for comprehensive adjustments and the consideration of potential confounders, thereby enhancing the reliability of the results. Early assessment using the Braden score can identify high-risk AP patients likely to experience poor outcomes, facilitating timely intervention and improved prognoses. Compared to other complex scoring systems, the Braden score offers advantages in simplicity, cost-effectiveness, and ease of calculation, making it applicable across various healthcare settings, including those with limited resources.

Despite providing valuable evidence for the prognostic significance of Braden scores in AP, certain limitations must be acknowledged. First, the single-center retrospective design restricts the ability to infer causality. Although we performed multivariate adjustments and subgroup analyses, residual confounders may persist, potentially undermining the prognostic outcomes. Thus, prospective multicenter studies are warranted. Second, due to data limitations, we were unable to conduct subgroup analyses based on AP etiology or obtain relevant imaging examination data. Future research should incorporate detailed etiological data. Third, our analysis focused solely on the initial Braden score at admission, with dynamic changes over time beyond the scope of this assessment. Further investigation into the prognostic value of dynamic Braden scores is necessary to clarify their clinical utility. Fourth, this study utilized data from a single-center ICU within the MIMIC-IV database, limiting the applicability of the findings to the AP population receiving intensive care. The generalizability to general wards or other healthcare settings requires further validation. Fifth, although the number of patients with missing Braden scores was negligible (*n* ═ 9, 0.45%), we cannot entirely dismiss the possibility that this minimal exclusion may introduce selection bias if the missing values correlate with unmeasured severity or frailty indicators. Lastly, 230 patients died within 30 days in this study, and 33 covariates were included in Model 3, yielding an events-per-variable (EPV) ratio of approximately 6.9, slightly below the conventional threshold of ≥10. While VIF-based exclusion methods were employed, potential overfitting risks remain, highlighting the need for validation in an independent cohort.

## Conclusion

This study expands the application of the Braden score in predicting outcomes for patients with AP, suggesting its utility as a straightforward, early supplementary indicator for risk stratification and identification of individuals at higher mortality risk. As this analysis is based on a single-center retrospective study, the findings are preliminary and require confirmation through future prospective multicenter cohorts to establish the clinical value of the Braden score as a simple bedside supplementary tool.

## Supplemental data

Supplemental data are available at the following link: https://www.bjbms.org/ojs/index.php/bjbms/article/view/13115/4024.
